# Limits of the detection of microplastics in fish tissue using stimulated Raman scattering microscopy

**DOI:** 10.1364/BOE.519561

**Published:** 2024-02-09

**Authors:** Moritz Floess, Marie Fagotto-Kaufmann, Andrea Gall, Tobias Steinle, Ingrid Ehrlich, Harald Giessen

**Affiliations:** 1 4th Physics Institute and Stuttgart Research Center of Photonic Engineering, University of Stuttgart, Pfaffenwaldring 57, 70569 Stuttgart, Germany; 2 Dept. Neurobiology, Institute of Biomaterials and Biomolecular Systems, University of Stuttgart, Pfaffenwaldring 57, 70569 Stuttgart, Germany

## Abstract

We demonstrate the detection sensitivity of microplastic beads within fish tissue using stimulated Raman scattering (SRS) microscopy. The intrinsically provided chemical contrast distinguishes different types of plastic compounds within fish tissue. We study the size-dependent signal-to-noise ratio of the microplastic beads and determine a lower boundary for the detectable size. Our findings demonstrate how SRS microscopy can serve as a complementary modality to conventional Raman scattering imaging in order to detect and identify microplastic particles in fish tissue.

## 1. Introduction

In recent years, the contamination of drinking water supplies and the food chain with micro- and nanoplastics has become an increasingly recognized problem. Enormous amounts of plastic waste worldwide find their way into the environment and ecosystems. Waste dumping into rivers constitutes the largest source of plastic waste in the oceans [1,2]. Over time, environmental weathering causes plastic parts to continuously fragment into smaller debris, so-called secondary microplastics, which can reach the micrometer or even the nanometer scale [3,4]. In addition, microplastic additives in cosmetic products, i.e., primary microplastics, find their way directly into local water supply systems [5–7]. Through uptake by, e.g., seashells or fish, these micro- and nanoplastic particles enter the food chain and may end up in our food, and finally, in our digestive system [8,9].

The exact physiological consequences of micro- and nanoplastics in the human body are still not clear and are currently under extensive investigation [10,11]. However, it is assumed that the potential health risks increase as the particle size decreases [12]. Below certain dimensions, nanoplastics may be able to traverse the intestinal barrier and enter the bloodstream [13]. In order to assess these potential health risks, in a first step, the detection and characterization of micro- and nanoplastics is necessary. Therefore, suitable detection and imaging methods to monitor the abundance of micro- and nanoplastics in the food chain are of high demand. Combined systems of dark-field imaging and spontaneous Raman spectroscopy, which are commercially available, are employed to screen drinking water supplies for contamination with microplastics [14,15]. However, conventional Raman micro-spectroscopy suffers from low acquisition speeds, which renders it unfeasible to scan entire samples without first determining the location of the plastic particles using an additional imaging modality, such as dark-field imaging. In order to overcome these speed limitations, stimulated Raman scattering (SRS) microscopy can be employed. SRS provides images with intrinsic chemical contrast, i.e., no additional labels are required, at up to video-rate acquisition speeds [16]. SRS micro-spectroscopy has been employed to estimate the relative abundance of synthetic microfibers in environmental samples [17]. Furthermore, SRS has been used to identify and distinguish microfibers of natural and synthetic origin from different geographical areas and environments, including microfibers from deep-sea sediments [18].

In this work, we investigate the detection of microplastics in fish tissue, which may ultimately pave the way to screen for microplastics contamination in fish populations. Hereby, microplastic beads serve as well-controlled model system for microplastic debris occurring in environmental samples. In particular, we determine the detection limit of polystyrene (PS) and polymethylmethacrylate (PMMA) microplastic particles under optimal conditions, i.e., in aqueous solution, using SRS microscopy in the C–H stretch spectral range. We demonstrate that the two different plastic compounds can be distinguished in fish muscle tissue based on their characteristic Raman responses. As for other types of tissue, muscle tissue contains the main chemical compounds (proteins, lipids, etc.), and thus, is well-suited to serve as test system to characterize the detection limits of microplastics. Furthermore, we show that microplastics can be detected and localized within bulk tissue employing the 3D imaging capability of our SRS system. Finally, we determine the limit for unambiguous detection and characterization of microplastic particles in fish tissue for our SRS microscope, which results in 1 µm particle size and a pixel integration time (*T*_int_) of 30 µs. Our findings can be extended to other plastic compounds, which occur in environmental samples.

Our SRS approach is intrinsically orders of magnitude faster than Raman micro-spectroscopy, due to the increased interaction cross-section. It is one of the tasks of this work to find out about the signal-to-noise ratio of SRS microscopy of micro- and nanoplastics in-situ in a relevant fish tissue environment.

## 2. Materials and methods

### 2.1. Laser system and SRS microscope

**Fig. 1.**
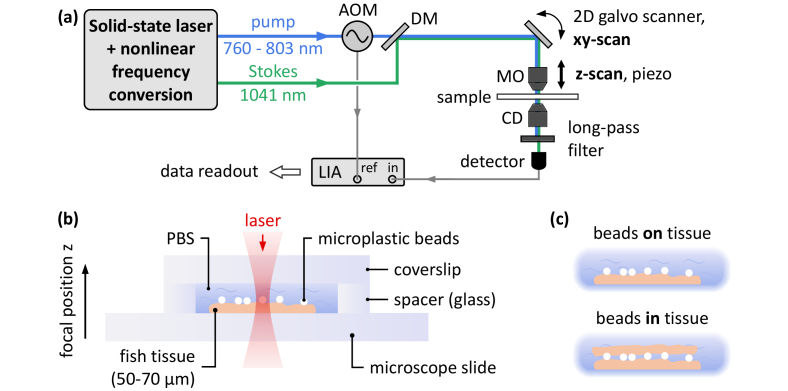
SRS microscope setup and sample geometry. (a) The pump and Stokes beams are provided by a solid state laser system (Yb:KGW oscillator: 8 W, 450 fs, 41 MHz, and 1041 nm) with subsequent nonlinear frequency conversion. SRG detection of the Stokes is employed, where an acousto-optical modulator (AOM) is used to modulate the pump beam. 3D scanning is realized using a 2D galvo scanner unit in combination with a piezo actuator, which enables z-scan capability. DM: dichroic mirror, MO: microscope objective, CD: condenser. (b) The fish tissue is kept in physiologically neutral phosphate-buffered saline (PBS). Microplastic beads are placed on top of the fish tissue. The microscope slide, the spacer, as well as the coverslip have a thickness of 170 µm. Laser illumination from the top, detection in transmission. (c) Microplastic beads may either be placed on top of one tissue slice, or may be sandwiched between two slices. The muscle structures of the stacked tissue slices are deliberately oriented in different directions.

Pump and Stokes beams are both provided from the same solid-state laser system, as depicted in Fig. 1(a). An 8-W Yb:KGW oscillator with a pulse repetition rate of 41 MHz serves as front-end and directly provides the Stokes channel at a fixed wavelength of 1041 nm. A part of the oscillator is used to operate an optical parametric oscillator [19] and a subsequent second-harmonic generation stage, which provides the tunable Raman pump with a wavelength in the range between 760 and 803 nm. Thus, the pump-Stokes detuning allows to access Raman bands in the range of 2850–3550 cm^−1^. As both channels are provided by the same front-end, their pulse repetition rate is intrinsically synchronized, and therefore, the system is free of relative pump-Stokes timing jitter. The pulse duration of ∼1 ps corresponds to a spectral bandwidth of ∼10 cm^−1^, which directly translates to the spectral resolution of this system. A modulation frequency of 2.6 MHz is chosen, where the relative intensity noise of the laser system enables shot-noise limited detection and electronic cross-talk is minimized. A home-built low-noise detector based on a transimpedance amplifier design (Texas Instruments, OPA843) is used for detection. A lock-in amplifier (Zurich Instruments, UHFLI) is employed for signal extraction.

**Fig. 2.**
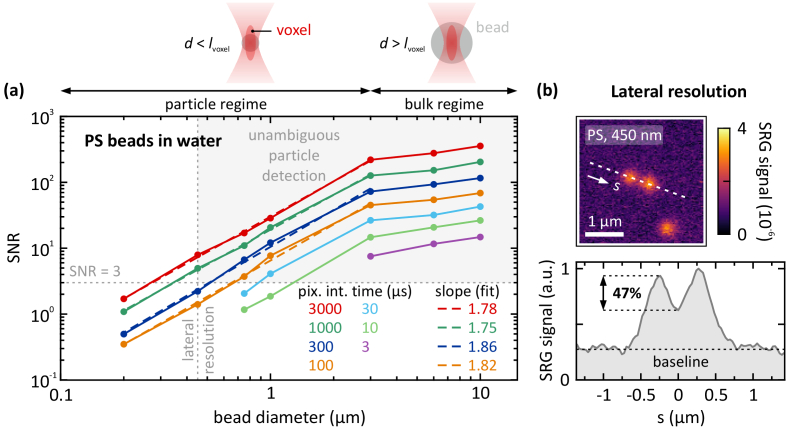
(a) Signal-to-noise ratio (SNR) depending on the diameter of the microplastic beads. PS beads in aqueous solution are used as reference system, in order to obtain an upper boundary for the achievable SNR. Given a priori knowledge about the microplastic particles and optimal conditions, beads down to 200 nm diameter are detectable. However, for unambiguous detection of microplastic particles with unknown dimensions, a minimum required SNR of 3 and a minimum size of > 0.45 µm (lateral resolution of the SRS microscope) are defined, as indicated by the gray shaded area. Hence, the detection limit is reached for 450-nm beads at a pixel integration time of 1000 µs, which yields a SNR of 5. The axial voxel size of *l*_voxel_ = 3 µm divides the range of particle sizes into two regimes, in which the SNR scales differently: particle regime for beads with *d* < *l*_voxel_ (SNR increases linearly on double-logarithmic axes), and bulk regime for beads with *d* > *l*_voxel_ (SNR almost saturates). Pump power: 10 mW, Stokes power: 9.5 mW. (b) 450 nm PS beads are imaged to determine the lateral resolution. Pixel integration time: 3 ms. The signal cross-section along the dashed line yields a modulation depth of 47% with respect to the noise baseline, therefore satisfying the Rayleigh criterion, which requires a modulation depth of 26.4% for two objects to be resolvable.

3D SRS imaging is realized using a 2-axis galvanometric mirror scanner (Cambridge Technology, 6220HM44B) for lateral scanning and a piezo actuator (Physik Instrumente, P-721-17) for axial scanning. Hereby, the positive z-direction points upwards, as indicated in Fig. 1(b). This convention is used for all following experiments. A microscope objective (Nikon S Plan Fluor ELWD 60x, NA = 0.7) with a long working distance of 1.8–2.6 mm using air as imaging medium is employed for laser illumination of the sample. An oil-immersion condenser (Nikon C-AA Achromat/Aplanat Condenser, NA = 1.4) collects as much of the transmitted Stokes light as possible. The condenser is used in air as immersion medium, as the image quality solely depends on the illumination with the microscope objective. The moderate numerical aperture of NA = 0.7 of the microscope objective provides the best compromise between maximum spatial resolution and the ability to image inside of scattering tissue. In fact, high-NA illumination is more susceptible to scattering and wavefront distortions, which eventually reduces the coherence at deeper positions in the tissue, and thus, deteriorates the SRS signal. The spatial resolution is given by the volume pixel element (voxel), in which \the SRS interaction takes place, i.e., the nonlinear 3D point spread function. The lateral resolution is experimentally determined to *w*_voxel_ = 450 nm, as depicted in Fig. 2(b). The axial resolution yields *l*_voxel_ = 3 µm, as we have reported in [20]. Therefore, particles which fall below the lateral resolution cannot be separated unambiguously from adjacent structures.

Apart from the SRS signal, the detected Stokes beam also carries the linear transmission properties of the sample. In particular, the DC voltage level at the detector is a direct measure thereof and is used to acquire linear transmission images at the Stokes wavelength.

### 2.2. Sample preparation

Our strategy was to apply microplastic beads with defined dimensions onto thin slices of fish tissue, in order to systematically study the detection capabilities and signal-to-noise ratios of our SRS microscope system.

In the first step, fresh mackerel fish muscle tissue was obtained from commercial vendors and transported to the laboratory. In the laboratory, tissue was cut into cubes of ∼1 cm^3^ and fixed in a fixation buffer containing 4% Paraformaldehyde (Roth, Germany) in phosphate buffered saline (PBS) of the following composition: 140 mM NaCl, 15 mM phosphate buffer (3 mM NaH_2_PO_4_, 12 mM Na_2_HPO_4_) (Roth, Germany) at a pH of 7.4 (pH was adjusted with NaOH or HCl). Initial fixation was carried out at room temperature (20–24 °C) for 1 hour, followed by post-fixation for ∼16 hours at 4 °C. Afterwards, the tissue was washed three times for 10 min with PBS and stored in PBS supplemented with 0.1% Sodium-Azide (NaN_3_, Sigma-Aldrich, Germany) at 4 °C until further processing.

In the second step, the tissue was sectioned into thin slices using a vibratome (Leica VT1200S, Leica Microsystems, Germany). The blocks of fish tissue were embedded into 2% agar (Agar-Agar, Roth, Germany) in order to stabilize the tissue during slicing. Tissue slices were cut at a thickness varying between 50 and 70 µm. The slices were collected and stored in PBS at 4 °C.

Figure 1(b) depicts the sample geometry. Glass spacers with a thickness of 170 µm were glued on top of the microscope slide to form a closed container for the tissue. The tissue slices were placed onto the microscope slide within the spacer containment. Diluted microplastic bead solutions (0.25% microplastic particles) were prepared in PBS from a 2.6% polystyrene (PS) bead stock solution (Polysciences, USA), and from a 10% polymethylmethacrylate (PMMA) bead stock solution (microParticles GmbH, Germany), respectively. 5 µl of the diluted microplastic beads solution were applied onto the tissue using a syringe. Depending on the experiment, a second tissue slice was sandwiched on top, as depicted in Fig. 1(c). The remaining containment volume was filled with PBS to ensure that no air bubbles were present. In the final step, a coverslip was placed on top and sealed using 2-component epoxy glue in order to prevent the sample from dehydrating.

## 3. Results

The signal-to-noise ratio (SNR) scales as $\text{SNR}\propto N^{1/2}$ , where *N* denotes the number of photons which are involved in the SRS interaction. However, we use an alternative form SNR = (µ_signal_ – µ_bg_)/σ_noise_, where µ_signal, bg_ and σ_noise_ denote the mean values of signal and background, and the noise standard deviation, respectively. This allows the direct assessment of the SNR as these quantities can be extracted directly from the resulting images. First, an upper boundary for the achievable SNR was determined. Therefore, PS beads in aqueous solution (deionized water) without any tissue involved were prepared. This provides a reference system with optimal conditions, i.e., a low background signal level µ_bg_, from which a benchmark for the achievable SNR can be deduced. Beads with diameters between 200 nm and 10 µm were imaged using the SRS microscope. A separate sample was prepared for beads of different diameters. For each bead diameter, a pixel integration time sweep between *T*_int_ = 100 and 3000 µs was carried out (for larger bead diameters *T*_int_ was varied down to 3 µs), and the resulting SNR was extracted from the SRS images, as plotted in Fig. 2(a).

The axial voxel size *l*_voxel_ = 3 µm divides the range of particle sizes into two regimes, where the SNR scales differently with the bead diameter. A linear behavior (note the double-logarithmic axes) is observed for this particle size range, which we define as the *particle regime*. For this regime, the fitted slope values range from 1.75 to 1.86, as indicated in Fig. 2(a). In contrast, for bead diameters *d* > *l*_voxel_ the SNR exhibits only a minor increase and almost saturates at values of ∼300 at a pixel integration time of 3 ms. At *T*_int_ = 3 µs the SNR reaches values of ∼10. We define this range as the *bulk regime*. Figure 2(b) depicts the cross-sectional signal of two 450 nm PS beads to assess the lateral resolution limit. The resulting modulation depth of 47% satisfies the Rayleigh criterion, which states that the intensity in the area between the overlapping Airy disks of two close-by objects has to be decreased by >26.4% [21–23]. Thus, the lateral resolution of the SRS system reaches ∼450 nm at *T*_int_ = 3 ms. For shorter integration times the lateral resolution degrades, as the noise level increases.

**Fig. 3.**
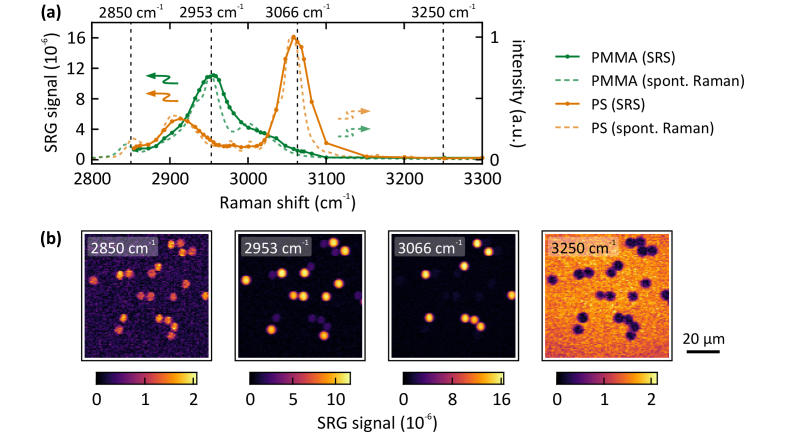
(a) Measured SRS spectra of PS and PMMA beads, both 6 µm diameter. The SRS spectra agree well with the literature spontaneous Raman spectra (dashed lines). (b) Exemplary SRS scans at the spectral positions indicated by the vertical dashed lines in (a). Pixel integration time: 3 ms, pump power: 6 mW, Stokes power: 10 mW.

As one of the benefits of SRS imaging is the intrinsic chemical selectivity, we measure the SRS spectra of PS and PMMA beads, as depicted in Fig. 3(a). The peak positions as well as the peak ratios agree well with the corresponding spontaneous Raman spectra. Due to the spectral resolution of ∼10 cm^−1^, sharp spectral features appear smoothed in the SRS spectra. Figure 3(b) depicts exemplary SRS scans at four different spectral positions. At the resonance of saturated lipids (2850 cm^−1^), PS and PMMA beads exhibit comparable residual signal levels. The PS beads show a remaining signal at 2953 cm^−1^. The highest chemical contrast is reached at 3066 cm^−1^. At 3250 cm^−1^, the SRS signal of the surrounding water exceeds the response of the beads.

**Fig. 4.**
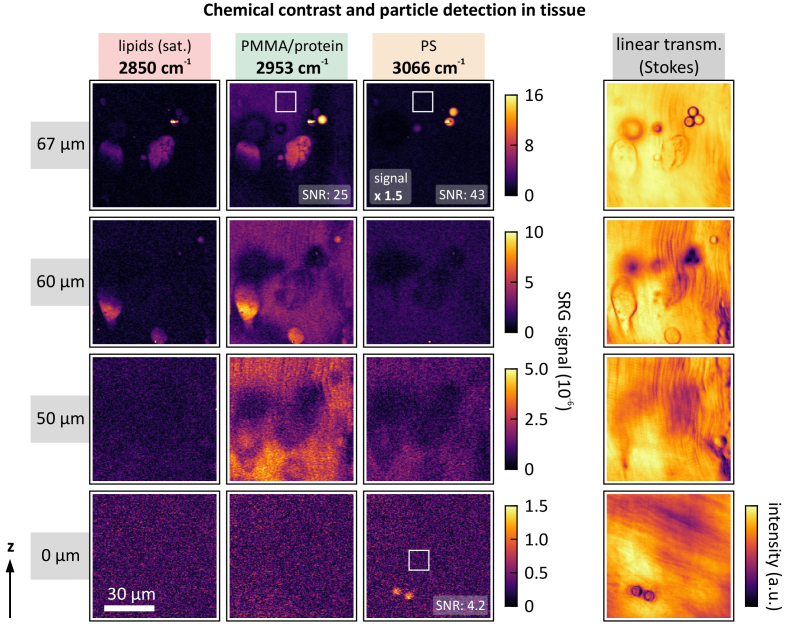
Chemical contrast between PS and PMMA beads in fish tissue environment. Bead diameter: 6 µm. Two tissue slices are stacked on top of each other, as depicted in Fig. 1(c). SRS images are acquired at 2850 cm^−1^ (saturated lipids), 2950 cm^−1^ (PMMA/protein), and 3066 cm^−1^ (PS) for four different z-positions. The corresponding linear transmission (Stokes channel) images are depicted on the right. Note the different orientation of the muscle tissue at z = 0 (at the interface between the tissue slices, Fig. 1(c)). PS and PMMA beads can be distinguished by their respective Raman resonances. Especially PS exhibits excellent contrast against the tissue environment. SNR values are determined for the brightest beads and the noise regions marked by the white squares, respectively. The three beads at *z* = 67 µm are located on top of the tissue. A laser-induced defect at one of the beads causes a strong spectrally independent signal, which arises from cross-phase modulation. In contrast, the PS beads at *z* = 0 µm are located within the tissue, i.e., in between two tissue slices. At this position, the SRS signal level drops by a factor of ∼15 as the laser propagates through ∼65 µm of tissue, resulting in a SNR of 4.2. Note the signal multiplication factor for the upper right image. Pump power: 7 mW, Stokes power: 9.5 mW, 150 × 150 px^2^, pixel integration time: 300 µs (1 ms at z = 0), pixel dwell time: 1 ms (3.3 ms at z = 0), acquisition time per image: 25 s (75 s at z = 0).

In the next step, we demonstrate chemical contrast between PS and PMMA beads of 6 µm of diameter in fish tissue environment, as depicted in Fig. 4. A bead cluster of both plastic types in tissue was imaged at their respective Raman resonances at 3066, and 2953 cm^−1^, respectively. Additionally, images at the Raman band of saturated lipids at 2850 cm^−1^ were acquired. As control measurements, linear transmission images at the Stokes wavelength were acquired, which reveal the cluster of beads as well. The PMMA beads are well visible at 2953 cm^−1^. Nevertheless, the PMMA Raman resonance overlaps with the protein resonance, which also highlights tissue structures, and therefore, limits the contrast of the PMMA beads against the background, yielding a SNR of 25. On the other hand, excellent contrast with almost no background signal is observed for the PS beads, resulting in a SNR of 43. In agreement with Fig. 3, the SRS signatures of both, PS and PMMA beads, almost vanish at the lipids resonance at 2850 cm^−1^, which demonstrates the chemically selective imaging capability of our SRS system. As expected, the bead cluster vanishes at deeper z-positions in the tissue, as evident from the scans at *z* = 50 and 60 µm.

Further down in the sample, two PS beads are revealed between the two tissue slices at *z* = 0. In this case, the signal level dropped by a factor of ∼15 compared to the signal originating from the beads on top of the tissue. An integration time of 1 ms (3.3 ms pixel dwell time) yields a SNR of 4.2. Again, the linear transmission image confirms the location of the beads.

**Fig. 5.**
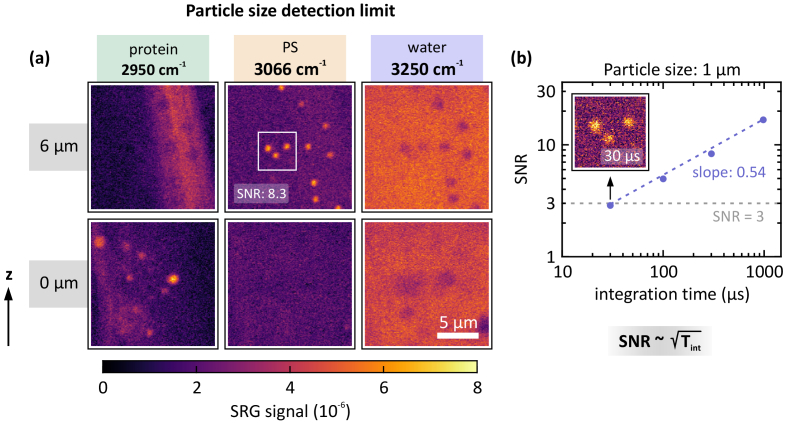
Detection of PS beads with 1 µm diameter on fish tissue. (a) SRS images are acquired at 2950 cm^−1^ (PMMA/protein), 3066 cm^−1^ (PS), and 3250 cm^−1^ (water) for two different z-positions. The 1-µm PS beads are clearly distinguishable. Pump power: 7 mW, Stokes power: 9.5 mW, 150 × 150 px^2^, pixel integration time: 300 µs, pixel dwell time: 1 ms, acquisition time per image: 25 s. (b) Signal-to-noise ratio (SNR) for varying integration times (*T*_int_). The white square at *z* = 6 µm indicates the analyzed region. The resulting slope agrees well with the expected square-root scaling. Inset: a SNR of 2.9 is reached at an integration of only *T*_int_ = 30 µs (pixel dwell time: 100 µs).

Figure 5(a) demonstrates the unambiguous particle size detection limit in fish tissue environment. In this experiment, PS beads with a diameter of 1 µm were placed on top of a slice of fish tissue. In order to account for the increased noise level at an integration time of only 300 µs, 1 µm beads are used to ensure unambiguous separation of the particles. The PS resonance (3066 cm^−1^) at *z* = 6 µm clearly reveals the beads. At the water resonance at 3250 cm^−1^, the beads appear as shadows surrounded by the water signature, while at the protein resonance (2950 cm^−1^), the beads are not detectable. As control measurement, images were acquired 6 µm below the bead location within the tissue (*z* = 0 µm), where the protein signature reveals the presence of tissue bulk material.

The SNR is analyzed depending on the pixel integration time, as depicted in Fig. 5(b). The resulting slope (note the double-logarithmic axes) of 0.54 agrees well with the expected square-root scaling behavior of the SNR. At an integration time as short as 30 µs (100 µs pixel dwell time), a SNR of 2.9 is reached, as depicted in the inset in Fig. 5(b).

## 4. Discussion

### 4.1. Detection limit of PS beads under optimal conditions

Generally, the detection limit for structural features such as microplastic particles using SRS microscopy is dictated by two factors. First, the signal-to-noise ratio (SNR) must be sufficient in order to obtain contrast against the noise floor. Secondly, the spatial resolution of the imaging setup determines whether individual particles can be spatially distinguished, and imposes a lower boundary on the minimum detectable particle size. Both conditions have to be met to ensure unambiguous particle detection. Consequently, particle sizes below the resolution limit cannot be directly assessed, even though the SNR level might be sufficient, and vice versa.

Nevertheless, given a priori knowledge about the microplastic particles, beads down to 200 nm diameter are detectable under optimal conditions, although they are well below the spatial resolution limit, as depicted in Fig. 2(a). Thus, in order to reach this sensitivity level, the bead dimensions must be known beforehand. Additionally, clustering of individual beads cannot be ruled out, as bead clusters may still be sized below the spatial resolution limit. The integrated signals of several potential clusters exhibit discrete differences in the signal level from which the number of beads within the clusters can be extracted. Furthermore, the detection with a SNR below 1 is possible only if the bead location is exactly known.

However, for unambiguous detection of microplastic particles with unknown dimensions and location, a minimum required SNR of 3 is defined and a minimum size of > 0.45 µm (lateral resolution of the SRS microscope) is necessary, as indicated by the gray shaded area in Fig. 2(a). As a result, we demonstrate that the detection limit for PS beads is reached, e.g., for a diameter of 1 µm with *T*_int_ = 30 µs, or for 450 nm beads with *T*_int_ = 1 ms, at the given laser power levels of 10 mW in the pump channel, and 9.5 mW in the Stokes channel. Increasing the pump power level would yield a linear increase in the SNR. However, the damage threshold of the sample imposes a limitation on the power level to ∼10 mW.

For beads with *d* < *l*_voxel_, the filling factor of the voxel scales with the bead volume (particle regime). Hereby, the number of excited molecular oscillators *N* increases with *N* ∼ *d*^3^. Since the SNR scales with *N*^1/2^, the SNR is expected to increase with *d*^3/2^. This is well reproduced by the mean slope value of 1.8 which is extracted from the linear fit curves. In contrast, for beads with diameters of > 3 µm (bulk regime), the voxel fully overlaps with the material independently of the bead diameter, and thus, the SRS signal almost saturates.

### 4.2. Chemical contrast and detection of microplastics in fish tissue

As depicted in Fig. 4, PS exhibits excellent contrast against the background, as the tissue does not respond at 3066 cm^−1^. Conversely, the PMMA resonance largely overlaps with the protein resonance at 2950 cm^−1^, and thus, the tissue response at this spectral position limits the contrast of the detected PMMA beads against the background. Therefore, SRS imaging in the fingerprint spectral region would be beneficial, as it provides improved chemical specificity. Nevertheless, PS and PMMA beads can be clearly distinguished, even if they conglomerate within the same cluster. Off-resonant imaging, in this case at the Raman band of saturated lipids at 2850 cm^−1^, indicates that the signal originates from SRS interaction rather than parasitic effects, such as cross-phase modulation (XPM).

SRS detection of microplastic particles within fish tissue is demonstrated at *z* = 0. Imaging of the 6 µm PS beads through the upper tissue slice still yields clear images with low distortions. However, deterioration of the SRS signal is observed, as evident from the signal level at *z* = 0. This is likely due to inhomogeneities in the tissue that cause wavefront distortions and scattering, both of which decrease the SRS efficiency, as it is a phase-sensitive process. In this specific case, the signal level drops by a factor of ∼15 after the propagation through ∼65 µm of tissue. Thus, while we demonstrate that SRS imaging can be employed to screen fish tissue for microplastic contamination, it is apparent that the penetration depth into the bulk tissue strongly depends on the local tissue composition and its overall transparency.

### 4.3. Particle size detection limit

As demonstrated in Fig. 2(a), unambiguous PS bead detection under optimal conditions demands a minimum bead diameter of 1 µm when using a minimum pixel integration time of 30 µs, which yields a SNR of 4.1. This could be replicated in fish tissue, where 1 µm PS beads, due to the low signal background level at 3066 cm^−1^, are detected with an only slightly reduced SNR of 2.9 at a pixel integration time of 30 µs, as depicted in the inset in Fig. 5(b). As this is right at the lower SNR limit of 3, it marks the detection threshold for PS in fish tissue and indicates that it is feasible to detect 1 µm particles under more realistic conditions.

Clearly, this assessment holds true for the specific case of PS microplastic particles. Other plastic compounds with different Raman response levels may exhibit deviating figures of merit, and thus, may be harder to detect. As in the case of PS, disjoint Raman resonances between the microplastic particles and the surrounding tissue are highly beneficial for optimized chemical contrast. As apparent from Fig. 4, PMMA microplastics detection in the C–H stretch spectral region suffers from the overlapping protein resonance, which highlights surrounding tissue as well.

### 4.4. Limitations of SRS microscopy for the detection of microplastics

The spatial resolution imposes a lower boundary on the minimum detectable particle size of ∼450 nm. Below that, the actual particle size cannot be assessed. Furthermore, in this particle size range the SNR drops with decreasing particle volume $\left(\text{SNR}\propto V^{1/2}\right)$ . Therefore, individual nanoplastic particles with dimensions significantly smaller than 1 µm cannot be detected using SRS imaging, unless several smaller particles are conglomerated within a cluster.

Furthermore, the penetration depth into the tissue we demonstrate is on the order of ∼65 µm. Although higher penetration depths may be possible, this would strongly depend on the tissue texture and composition. On the one hand, limited tissue transparency hinders imaging deep inside the tissue without the laser power exceeding the tissue damage threshold. On the other hand, a loss of coherence is induced by scattering. Especially inhomogeneous tissues could deteriorate the wavefronts of the incident laser beams, which in turn decreases the SRS interaction efficiency, as observed in Fig. 4 at *z* = 0. Hence, careful tissue preparation is necessary prior to the imaging process. In particular, thin slicing may be required, as demonstrated in this work.

SRS imaging is well suited for imaging of biological systems with high resolution and detail within fields of view below 1 mm^2^. However, large area or even large volume scans using SRS microscopy would require image stitching, and as a result, the overall acquisition time would increase drastically. Therefore, the region of interest within a large sample should be known or determined in advance to enable directed SRS imaging within this region. Consequently, unbiased monitoring of large volumes for traces of microplastics based on SRS imaging remains not yet feasible.

### 4.5. SRS imaging as complementary technique to conventional Raman imaging

Conventional Raman imaging provides the entire Raman spectrum per pixel with high spectral resolution, which renders this technique ideal for the identification of unknown compounds [24,25]. SRS in its original scheme, on the other hand, intrinsically probes one resonance at a time only. In principle, spectral multiplexing is possible to a certain extent, but it requires increased technical effort, and thus, adds cost and complexity [26–28]. Additionally, the spectral resolution of SRS is intrinsically limited, as pulse duration requirements impose a limitation to narrow-band detection.

However, owing to its significantly enhanced interaction cross-section, SRS enables much faster image acquisition than conventional Raman imaging [29]. In order to reduce the acquisition time, conventional Raman imaging is combined with dark-field imaging, which is employed to detect the location and the size distribution of microplastic particles in advance [15,30]. With that, the subsequent Raman spectroscopic measurement can be used specifically at the location of interest only. However, this combined technique can only be employed in conditions, where the microplastic particles can be isolated from their surrounding medium and prepared accordingly, such as filtered particles for monitoring of microplastic contamination in drinking water.

SRS imaging is a superior choice for tissue screening for microplastic contamination, as it does not require filtration and extraction of the plastic particles by elaborate chemical treatment in advance [31,32]. Additionally, SRS microscopy reveals the location of the microplastic particles in the surrounding tissue, which will be useful to unravel precise patterns of accumulation in diverse tissue types and organisms, and thus, enables investigations into its physiological consequences in the future.

## 5. Conclusion

We have demonstrated microplastics detection in fish tissue based on SRS spectral imaging and determined an upper boundary for the achievable signal-to-noise ratio for PS microplastic beads. Considering the spatial resolution of the SRS microscope, we have defined the requirements on unambiguous microplastic particle detection. For PS particles the detection limit is reached for a diameter of 1 µm and a pixel integration time of 30 µs at 10* *mW of pump power. Other plastics compounds, such as PMMA, exhibit lower contrast against the tissue background due to spectrally overlapping Raman responses.

Extending the wavelength tuning range to the fingerprint spectral region would enable access to Raman bands with increased specificity, and thus, to increase the contrast between microplastics signatures and the surrounding tissue.

The sensitivity could be further enhanced by utilizing a high-NA water immersion microscope objective for the laser illumination of the sample to increase the SRS signal level as well as the spatial resolution. However, the imaging performance within scattering and absorbing tissue would deteriorate, and therefore, limit this approach to microplastic detection close to the tissue surface.

The SRS image acquisition speed could be further enhanced by increasing the modulation frequency up to 20.5 MHz. Such Nyquist modulation schemes have been reported for SRS [33,34], as well as for stimulated emission microscopy [35]. This could be realized using an extended-cavity design for the fiber-feedback optical parametric oscillator in combination with pump modulation to provide maximum modulation depth [36]. However, the expected increase in the noise level at higher acquisition speeds would limit the detection to larger microplastic debris on the order of 10 µm diameter.

In conclusion, SRS spectral imaging is an ideal complementary modality to conventional Raman scattering imaging for the detection and identification of microplastic particles. In particular, SRS is superior for imaging microplastics contamination in tissue, where the particle extraction from the tissue prior to chemical identification and particle size assessment is unfeasible. Currently, there is no single detection technique available, which combines all requirements, i.e., high acquisition speed, chemical specificity, high 3D spatial resolution, large-area fields of view, and large penetration depth. Consequently, for every specific use case, the detection methodology has to be chosen accordingly. We believe that 3D SRS spectral imaging holds the potential to be an important tool for the assessment of possible health risks imposed by microplastics in our food chain.

## Data Availability

Data underlying the results presented in this paper are not publicly available at this time but may be obtained from the authors upon reasonable request.
